# Comorbidity of headache in children and adolescence

**DOI:** 10.1186/1129-2377-14-S1-O8

**Published:** 2013-02-21

**Authors:** Çiçek Wöber-Bingöl

**Affiliations:** 1 Medical University of Vienna, Austria

## 

In its first part, this presentation will cover a short review of the features of migraine and tension-type headache in children and adolescents, which is a fascinating chapter and will especially focus on migraine aura, confusional migraine and chronic headache.

It the second part somatic and psychiatric comorbidities in children and adolescents with migraine will be reviewed covering asthma and allergies, vascular disorders, sleep disturbances, depression and anxiety as well as Tourette syndrome and attention deficit hyperactivity disorder.

